# Hemp (*Cannabis sativa* L.) Seed and Co-Products Inclusion in Diets for Dairy Ruminants: A Review

**DOI:** 10.3390/ani11030856

**Published:** 2021-03-17

**Authors:** Lucia Bailoni, Elisabetta Bacchin, Angela Trocino, Sheyla Arango

**Affiliations:** Department of Comparative Biomedicine and Food Science (BCA), University of Padova, Viale dell’Universitá 16, 35020 Legnaro, PD, Italy; elisabetta.bacchin.1@studenti.unipd.it (E.B.); angela.trocino@unipd.it (A.T.); sheylajohannashumyko.arangoquispe@phd.unipd.it (S.A.)

**Keywords:** hemp, dairy ruminants, milk yield, milk composition, fatty acid profile

## Abstract

**Simple Summary:**

Hemp (*Cannabis sativa* L.) is an annual herbaceous plant, globally distributed and cultivated in the past as an important source of fiber. Recently, the interest in hemp cultivation has significantly increased, considering its positive impact on the environment and the production of feed and food of high nutritional value. The authorized hemp varieties are registered in the EU’s Common Catalogue of Agricultural Plant Species and have content in psychotropic 9-tetrahydrocannabinol (THC) less than 0.2–0.3%. In this review, the chemical and nutritional values of hemp are presented and the effects of inclusion of the hempseed, and products obtained by the processing of seed (co-products), in diets for dairy ruminants (i.e., cows, ewes, goats, and buffaloes) are discussed. Hemp supplementation could be a good feeding strategy to improve the bioactive compounds in milk and derivatives: the content of n-3 fatty acids and isomers of conjugated linoleic acid, substances beneficial to human health, increased in milk and cheese obtained with hemp addition. However, up to now, few publications do not allow to suggest the optimal dosage of the co-products for the different species. In addition, no experiments are published on the use of whole plants as forage for dairy ruminants.

**Abstract:**

Recently, hemp (*Cannabis Sativa* L.) was rediscovery as a plant that offers a wide variety of applications (textile, pharmaceuticals, construction, etc.), including also the use in animal and human nutrition. The inclusion of whole seeds and co-products obtained by processing of seeds (cake, meal, and oil) in the diets of farm animals can allow the transfer of bioactive substances to human food. Few publications are available on the use of hemp in dairy ruminants but some authors reported a positive effect on the fatty acids profile of milk and cheese with an increase of n-3 fatty acids and c9,t11 conjugated linoleic acid. The protein content, amino acids profile, and rumen undegradable protein (RUP) of hempseed and co-products of hemp appear interesting and suitable for ruminant nutrition. Negative effects of anti-nutritional factors (i.e., phytate) are not observed. However, the researches on the effects of the use of hempseed and co-products in diets for dairy ruminants do not allow to suggest optimal levels of inclusion. In addition, no data are published on the use of whole or part of the hemp plant as forage, as another possibility to use the hemp in the perspective of the circular economy.

## 1. Introduction

The consumption of animal products (meat, milk, and eggs) is growing globally mainly due to an increase in world population, greater incomes, and urbanization [1]. The growing demand for livestock products can have an undesirable impact on the environment, considering, in particular, low energy conversion ratio from feed to food and the high requirements of land and other input (i.e., water, nitrogen) to produce the feed for animals. Ruminants are animals with a lower efficiency to convert the energy of feed in food considering the losses due to rumen fermentation processes. On the contrary, ruminants play an important role in the bio-economy by converting food not edible by humans (i.e., forages, crop residues, and agricultural by-products) into high nutritional value food [2,3].

On this basis, alternative plants have been recently rediscovered and reintroduced on the agricultural surfaces by exploiting (i) their higher resistance to the adverse conditions (i.e., drought, pathogens); (ii) their role as phytoremediation and soil revitalization [4]; and (iii) their lower nutritional requirements compared to traditional sources of energy and protein in ruminant feeding (mainly corn meal and corn silage, soybean meal, etc.). The hemp plant (*Cannabis sativa* L.) is undoubtedly one of the most cultivated plants throughout history in the world.

The surface of hempseed in Europe, estimated by European Industrial Hemp Association (EIHA) [5], was about 50,081 hectares in 2018 with an increase of 3.3%, 70%, and 614% compared with 2017, 5-years average and 1993, respectively. The major producers in the world are Canada and USA with an estimated 315,000 and 1160 hectares respectively, as reported by Semwogerere et al. [6].

In the past, hemp has been cultivated primarily to obtain fibers from the stem [7,8]. The seeds have traditionally been used for therapeutic purposes and in pharmaceutics and chemistry [9], and the cannabinoid-containing flowers have been utilized for medicinal, spiritual/religious, and recreational purposes [10].

In Europe, the varieties allowed to be cultivated must be listed in the European Union (EU) Common Catalogue of Varieties of Agricultural Plant Species. The varieties must contain <0.2% delta-9-tetrahydrocannabinol (THC, in dry matter basis), which is the main psychoactive substance [11]. The interest to this plant is mainly oriented to produce seed for human and animal nutrition, shives for construction (green building) and animal bedding, and fibre for textile and paper industry (“industrial hemp”). In dairy ruminant nutrition, hempseed and derivatives (oil, cake and meal) can be used as a supplement in feed mainly as sources of essential fatty acids and essential amino acids [12].

The aim of the present review paper was to report an update of data on the chemical and nutritional characteristics of hempseed and derivatives and a state of the art on the researches on the use in dairy ruminant feeding, considering their effects on the milk yield and quality.

## 2. Chemical Composition and Nutritive Value of Hempseed and Derivatives

### 2.1. Chemical Composition and Nutritive Value of Full-Fat Hempseed

The whole (full-fat) hempseed (HS) can be used as fed in the animal feeding or after the treatments to removal lipid components using cold mechanical pressing in order to obtain hempseed cake (HC) or, less frequently, by chemical extraction using organic solvents to obtain hempseed meal (HM). Some authors use the term “hemp meal” or “hemp flour” to indicate the product obtained by the mechanical extraction because the cake is often subjected to grinding and then it is in the form of powder. In this paper, “hempseed cake (HC)” will be used for both of these products.

Hempseed varieties, which are generally used for animal nutrition, are considered THC free; however, some studies have reported traces of THC present in the seed sample probably because it was contaminated with plant debris [13].

In Table 1, data of the chemical composition of the full-fat hempseed reported in the literature are shown. The expected differences of the chemical composition in the published studies are due to the effect of variety/cultivar, preliminary treatments (i.e., decortication), different pedological and climatic situations, and agronomical practices. The ripened seed of hemp is an excellent protein source in animal feeding (on average 24.8 ± 2.0% on dry matter, DM). A similar value of crude protein (25% on DM) for hempseed was reported by European Food Safety Authority (EFSA) [11]. Considering other protein sources, largely diffused in animal feeding, the hempseed can be located as an intermediate crude protein (CP) source between soybean (39.2 ± 5.4% on DM) and sunflower seeds (19.2 ± 4.2% on DM) [14]. The average percentage of lipids in hempseed is very high and results in 30.9 ± 4.2% on DM. Lower values were found by Arango et al. [15], considering six different varieties, cultivated in the north of Italy (province of Rovigo) in 2019.

The neutral detergent fiber (NDF) content (Table 1) showed a large variability among the authors, ranging from 29.7–37.2% on DM. Only four publications reported the energy value of hempseed, resulting on average 2422 ± 97 and 946 ± 117 kJ/100 of DM respectively for gross and net energy for lactation in sheep [16].

**Figure 1:**
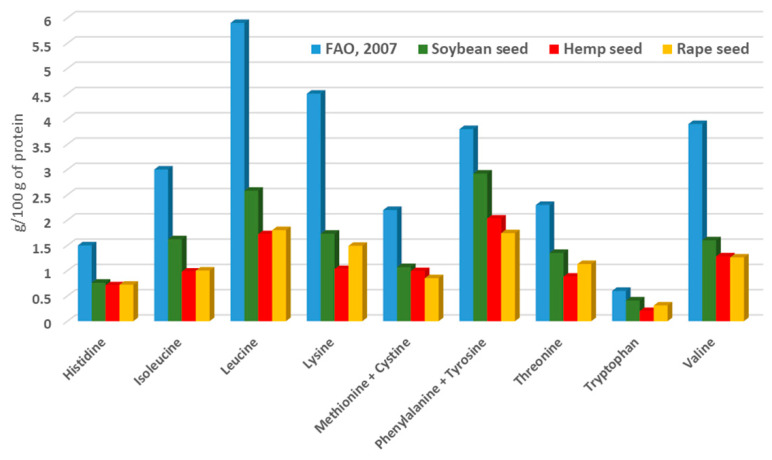
Content of essential amino acids (g/100 of protein) in soybean, hemp, and rape seed [9] in comparison with the reference pattern of FAO/WHO/ONU [2] for human nutrition.

Identification and characterization of hempseed proteins showed that edestin, rich in valuable amino acids (as glutamic acid and aspartic acid), is the main protein component in isolate hempseed protein fraction [24]. Another protein structure, rich in methionine and cystine, was found in hempseed and, subsequently, characterized as an albumin protein family member [25]. Callaway et al. [9] reported, for the first time, the amino acidic profile of hempseed (cultivar Finola) in comparison with the other protein sources. The composition of essential amino acids of hempseed, soybean, and rapeseed [9] compared with the reference pattern recommended by FAO/WHO/UNU [26] in human nutrition, is presented in Figure 1. The contents of the sulphur-containing amino acids and histidine of hempseed are very similar to those of the other two protein sources. Only levels of lysine, threonine, and tryptophan are lower in hempseed compare to soybean and rapeseed. Considering the reference pattern of FAO/WHO/UNU [26] for adults, the limiting amino acid of hempseed is lysine (chemical score: 0.23).

Hempseed contains anti-nutritional compounds that reduce the absorption of protein and micronutrients. In particular, the phytate (inositol hexaphosphate) content in the seeds and cake of hemp can be over 5% [27]. The absorption of mineral elements and vitamins can be reduced by phytic acid, during the gastrointestinal passage, producing an insoluble final product [28]. Therefore, an additional amount of microelements is needed to maintain the efficiency of the metabolic processes that support growth, development, and a correct functioning of the organism [29]. Reggiani and Russo [30] observed that the replacement of 6.4% (on DM basis) of corn and soybean with hempseed or flax, maintaining the diets isonitrogenous, can increase the availability of iron in Alpine lactating goats. The authors speculate that some substances (i.e., inulin) contained in hemp or flax seeds can stimulate the absorption of iron.

### 2.2. Chemical Composition and Nutritive Value of Hempseed Meal

After oil extraction, the hempseed cake (HC) can be used as optimal protein source for dairy ruminants. The chemical composition of HC has been reported by several authors (Table 2). As expected, crude protein content increases in HC in comparison with hempseed, and the average value is 34.3% on DM. As other oilseeds, cold mechanical extraction of seed produces a cake that is higher in oil compared with the corresponding chemically obtained meals. The method of extraction is very important not only to obtain an oil of good quality but also to have a high oil yield [31,32]. The percentage of residual oil in the cake is 11.7–12.5% on DM for all authors; only Silversides [20] found a higher concentration of lipids (17.9% on DM). The content of fiber fractions increases in the hemp cake in respect to hempseed (about +27% and +42% for NDF and ADF, respectively).

### 2.3. Chemical Composition and Nutritive Value of Hempseed Oil

The quality of the oil obtained by chemical extraction is lower than that obtained by mechanical extraction. For this reason, hempseed meal is used mainly in the industrial processes (lubricants, detergents, paints).

In Table 3 is shown the fatty acid profile of whole hempseed (HS), hemp cake (HC) and hemp oil (HO) reported in the literature in order to compare the composition of fatty acids (FA) in the different products.

The contents of saturated FA (SFA) were very variable within the different products (from 8.2 to 14.5; from 7.7 to 13.1, and from 7 to 11.6 % of total FA for whole hempseed, cake and oil, respectively). In hempseed products, palmitic (C16:0) and stearic (C18:0) acids represent the higher percentages of SFA (on average 65% and 24% respectively). As known, these long-chain SFA, if consumed in excess, have been associated with increased cardiovascular disease risk in the human population [36,37].

The average values of the percentages of mono-unsaturated FA (MUFA) in the three products are very similar (13.4, 12.5, and 13.0% of total FA for hemp seed, cake, and oil, respectively). However, the variability within each group is very high, especially in oil (from a minimum of 9.0 to 20.7% of total FA). The oleic acid (C18:1) represents a very high percentage (from 93 to 98% of total MUFA).

As shown in Table 3, the sum of polyunsaturated fatty acids (PUFA) of hemp products is around 75%, and this value is reported as a mean by other authors [9,38,39]. The differences of single PUFA among the three products are very small but, within group, the variability is high, especially for alpha linoleic acid (ALA) in whole hempseed (from 12.98 to 22.4% of total FA). Over 70% of the PUFA are linoleic acid (LA; 18:2 n-6) and ALA (18:3 n-3) [40]. Small amounts of gamma-linolenic acid (GLA; 18:3 n-6) and stearidonic acid (SDA; 18:4 n-3), the biological metabolites of LA and ALA, respectively, were found by some authors (on average 4 and 2% of total FA) [41]. In all publications, the n-6/n-3 ratio is lower than 5:1, which has been claimed as ideal for humans [41,42].

## 3. Use of Hempseed and Derivatives in Dairy Ruminants

### 3.1. Use of Hempseed and Derivatives in Dairy Cows

The interest in the development of different feeding strategies to improve the chemical-nutritional properties of dairy milk and milk products, assuming that nutrition can influence milk composition in ruminants, has grown in the last years [48,49,50,51,52,53]. Considering the high level of n-3 and n-6 fatty acids and the optimal n6/n3 ratio in hempseed, an increase of these PUFA could be expected in milk and derivatives. However, no papers are available to date on the effects of hempseed cake inclusion in the diet of dairy cows on fatty acid profile of milk and derivatives.

There is only one published paper on the use of hempseed or its co-products in dairy cows. Karlsson et al. [33] evaluated the effects of increasing the proportion of hempseed cake (HC) in the diet of dairy lactating cows on milk production and composition. Four experimental diets (based on a ratio of 494:506 g/kg of DM between silage and concentrate mixture) were formulated to contain increasing concentrations of HC: 0 (HC0), 143 (HC14), 233 (HC23) or 318 (HC32) g/kg of DM. No effects in DM intake but significant linear increases in CP, fat, and NDF intakes were observed with the increase of the proportion of HC in the diets. Increasing HC dietary levels resulted in significant quadratic effects on the milk yields and energy-corrected milk, with the highest value for the HC14 group (Table 4). The milk protein and fat percentage decreased linearly (*p* < 0.05) with the increasing of HC in the diet. Furthermore, there was a significant (*p* < 0.001) linear increase in milk urea concentrations with the enhancement of HC inclusion due to the increase of CP intakes. A linear decrease in CP efficiency (milk protein yield/crude protein intake) was also observed. The best and maximum suggested level of HC inclusion in this experiment was 143 g/kg DM.

Mustafa et al. [54] determined the DM and CP in situ degradability in two non-lactating rumen fistulated cows of four different protein sources (hemp, borage, canola, and heated canola meals). The results showed that hemp meal resulted in an excellent natural source of rumen undegradable protein (RUP) (774 g/kg of CP), equivalent to heat-treated canola meal but higher than borage and canola meals.

In conclusion, further studies are required to determine the effects of including HC in dairy rations, suggesting to maintain the diets as isoenergetic and isonitrogenous, modifying the proportion of the other ingredients. In addition, the nutritional value of milk and derivatives (i.e., fatty acids profile, vitamins, bioactive substances) could be determined to know the possible nutraceutical effects of hempseed meal.

### 3.2. Use of Hempseed and Derivatives in Dairy Ewes

Ewes milk would naturally have a high content in substances beneficial to human health, such as n-3 fatty acids (FAs) and conjugated linoleic acid (CLA). The n-3 FAs, especially eicosapentaenoic acid (EPA, C20:5 n-3) and docosahexaenoic acid (DHA, C22:6 n-3), can reduce the risk of cardiovascular diseases and in experimental animals, c9,t11 CLA has been proved to possess anticancer and anti-atherosclerotic effects, as well as anti-obesity activities [55]. As above reported, to increase the concentration of PUFA in milk, different sources of unsaturated plant lipids (i.e., linseed, soybean, safflower, and sunflower) could be included successfully in the diet [56,57]. The disadvantage of milk enriched with PUFA is the possibility of oxidation owing to its high content of double-bonded molecules, which are prone to oxidation [58]. The delicate balance between anti- and pro-oxidative processes in milk is influenced by different factors such as ruminant feeding, degree of unsaturated fatty acids, contents of transition metal ions and antioxidants such as tocopherols and carotenoids [59].

In this context, Mierlita et al. [18] carried out an experiment using 30 Turcana dairy sheep divided into three groups consisting of a control diet (C diet) based on hay and supplemented by mixed concentrates and two experimental diets designed to provide the same amount of fat using hempseed (180 g/d) (HS diet) or hempseed cake (480 g/d) (HC diet). The three diets were isoenergetic and isonitrogenous, and the two diets with hemp had the same amounts of PUFA. Hemp (HS and HC diets) increased milk yield and milk fat content but decreased milk lactose (Table 5). The hemp feeding increased the PUFA content (especially n-3 fatty acids) in ewes milk and improved the n-6/n-3 ratio. Total CLA content doubled in the milk of the ewes that received hempseed and increased by 2.4 times with the hemp cake inclusion (Table 6). The alpha-tocopherol and antioxidant activity increased using hempseed in the diets, reducing the risk of lipid oxidation in raw milk.

Traditionally, ewes on farms are fed indoor or often on part-time grazing during much of the lactation period. During this period, the c9,t11 CLA and n-3 FA contents in milk are lower than that observed during grazing [60]. Mierlita et al. [19] studied the effects of a part-time grazing system or indoor feeding and the supplementation of hempseed in the diet on milk yield and quality, FA profile, and health lipid indices in the raw milk of dairy ewes. Forty ewes were used in this 10-week experiment and were divided into four groups: indoor feeding system with and without hempseed and part-time grazing with and without hempseed. Feeding with the addition of hempseeds significantly increased milk fat content and fat yield (Table 5). Milk protein content was not affected by dietary treatments. Hempseed supplementation increased the content of total PUFA, n-3 and n-6 fatty acids. In the indoor feeding system, the CLA content doubled with the hempseed addition (1.13 vs. 2.29% of total FA) but increased also in the milk of grazing sheep (+37%) (Table 6). As known, the availability of precursors (i.e., linoleic acid) for ruminal bio-hydrogenation and synthesis of CLA is high at pasture when the animals were fed fresh forage [61].

Ianni et al. [50] evaluated the effects of a diet enriched with hempseed (5% on DM basis) on the chemical characteristics of milk and cheese from 32 half-bred dairy ewes. The enrichment of dairy ewes’ diet with HS increased the lactose concentration from 4.69% to 5.84% but no significant differences were observed in milk fat, protein, casein, and urea (Table 5). In addition, no changes were detected in total fat, protein, and ash in derived cheeses. During the experiment reported by Ianni et al. [50], the first RNA sequencing of the whole blood transcriptome on ewes of the two experimental groups (0 and 5% of hempseed on DM) was described by Iannaccone et al. [13]. Hempseed supplementation positively affects the pathways related to energy production in lactating ewes. This condition could also be potentially beneficial to increase the resistance to adverse climatic conditions such as low temperature.

A digestibility experiment on sheep was conducted by Mustafa et al. [54] using hemp meal (5.2% of lipids on DM) at different levels of inclusion (0, 50, 100, 150, 200 g/kg of DM) in replacement of canola meal, maintaining isonitrogenous diets, based on barley. Voluntary DM intake was not affected by the hemp meal inclusion levels. Total tract DM and organic matter digestibility values were similar across treatments, suggesting that digestibility of hemp meal is equal to that of canola meal. The authors concluded that the hemp meal can be used up to 20% on DM with no detrimental effects on nutrient utilization by sheep.

### 3.3. Use of Hempseed and Derivatives in Dairy Goats

Goat milk has high concentrations of caproic (C6:0), caprylic (C8:0), and capric (C10:0) acids, known to exhibit antiobesity and antidiabetic properties [62]. Also in dairy goats, the interest of modulating milk fat composition by diet leads to the supplementation with feed sources rich in PUFA as an efficient way to modify milk FA profile. The oils extracted by oleaginous seeds can directly affect the fatty acid composition of milk and derivatives but could also have negative effects in terms of animal health status and, in particular, on the efficiency of the rumen microorganisms.

Cozma et al. [47] have evaluated the effect of a diet supplemented with hempseed oil in Carpathian goats during 31 days of experiment. No significant changes of milk yield were observed for ewes receiving the hempseed oil supplementation. Fat content increased significantly during the trial in milk produced by goats receiving hemp oil in comparison with the control group. The increase of milk protein content, due to the hemp oil addition, is significant just until day 15 of the experiment and then values remain the same (Table 7).

Cremonesi et al. [63] carried out an experiment to evaluate the effects of the inclusion of 9.3% on DM of linseed or hempseed in diet for Alpine lactating goat. The milk yield was unaffected by dietary treatment but linseed and hempseed supplementation significantly increased the milk fat content. No differences were detected in milk protein, lactose and urea concentration (Table 7).

Cozma et al. [47] found a significant increase of the PUFA concentrations (+45%) in milk produced by goats supplemented by hempseed oil, without an effect on n-3 fatty acids content. The content of cis-9, trans-11 CLA increased on average by over four times, reaching the peak during the second week of oil supplementation but then decreasing from the third week (Table 8). This transitory effect could be due to an adaptation of the rumen microorganisms to oil supplementation. Hemp oil inclusion had no effect on cholesterol concentration in milk (Table 8), even if plasma cholesterol concentration increased in the ewes fed with oil supplementation. The lack of a relationship between plasma and milk cholesterol concentration could be explained considering that a low proportion of the total milk cholesterol is derived from mammary de novo synthesis. In dairy cows, about 80% of the cholesterol in milk originates from the uptake of serum cholesterol obtained through hepatic synthesis [64]. The overall results of Cozma et al. [47] suggest, for the first time, that beneficial effects on human health can be obtained in goat milk with the inclusion of hempseed oil in the diets.

### 3.4. Use of Hempseed and Derivatives in Buffaloes

In several countries, buffaloes are important species for the production of milk and derivatives for human consumption. There are not any studies related to hemp as feed for improving buffalo milk. Only one published study [65] reported, in the north of Pakistan, possible exposure to delta-9-tetrahydrocannabinol (THC) by the children consuming buffaloes milk. In this region, buffaloes graze in natural pastures, where *Cannabis sativa* L. with high levels of THC grows spontaneously and higher concentrations of THC metabolites were found in buffaloes milk. As above reported, in EU countries, the hemp varieties allowed for cultivation are registered in the EU’s Common Catalogue of Agricultural Plant Species and are characterized by THC value less than 0.2–0.3% [11].

EFSA [11] recommended introducing a maximum THC content of 10 mg/kg to hempseed-derived feed materials to avoid risks for human health due to consumption of food of animal origin.

## 4. Conclusions

The chemical and nutritional characteristics of hempseed and hempseed derivatives (cake, meal and oil) are updated in the first section of this review. Protein content, aminoacids profile, and ruminal undegradable protein (RUP) make these products suitable for inclusion in ruminant diets. In addition, the fatty acid composition of hemp oil allows to transfer the PUFA and, in particular, n-3 fatty acid to the milk of dairy ruminants, as reported by several authors. Up to now, few publications are available on dairy ruminants to suggest the optimal dosage of hempseed or derivatives in the different species. No information about the use of the whole plant or the different botanical fractions (i.e., leaves) is published.

## Figures and Tables

**Table 1:** Chemical composition (% on DM basis) of full-fat hempseed.

References	[17] ^1^	[18] ^2^	[19]	[20]	[15] ^3^	[9]	[21] ^4^	[22] ^5^	[23]	Mean ± SD
DM, %	91.0	88.2	91.2	93.4	94.8	93.5	89.7	93.8	91.3	91.9 ± 2.2
Ash	4.9			5.8	4.7	6.0		5.5		5.4 ± 0.6
Crude protein	25.3	25.7	24.9	24.9	21.8	26.5	21.3	25.6	27.4	24.8 ± 2.0
Ether Extract	33.9	31.6	32.7	33.2	23.5	38.0	27.7	29.2	28.4	30.9 ± 4.2
Total dietary fiber (TDF)						27.6				
NDF	37.0	33.4	29.7	37.2				35.7	33.4	34.6 ± 3.1
ADF ^6^		23.2	21.3					27.8	23.3	24.1 ± 3.3
Gross Energy ^7^				2490		2353				2422 ± 97
Net Energy ^7,8^		1029	863							946 ± 117

^1^ Decorticated seed, ^2^ Cultivar Armanca, ^3^ Average of 6 cultivars, ^4^ Cultivar Jubileu, ^5^ Average of 10 cultivars, ^6^ Acid detergent fiber, ^7^ Energy is expressed as kJ per 100 g of DM, ^8^ Net Energy for lactation (sheep), estimated according to INRA [16].

**Table 2:** Chemical composition (% on DM basis) of hempseed cake (obtained by cold oil pressing).

References	[9] ^1^	[18] ^2^	[33] ^1^	[20] ^3^	[15] ^4^	[34]	Mean ± SD
DM	94.4	89.4	93.7	91.4	93.8	89.2	92.0 ± 2.3
Ash	7.6		6.7	7.9	5.8	6.5	6.9 ± 0.9
Crude Protein	35.5	33.4	34.4	33.6	31.4	37.7	34.3 ± 2.1
Lipids	11.8	11.7	12.4	17.9	12.5	9.6	12.7 ± 2.8
NDF		43.6	39.3			44.2	43.1 ± 2.6
ADF		36.2	32.1				34.2 ± 2.9
Gross Energy ^5^	1801			2319			2060 ± 366
Metab. Energy ^5,6^			950			1256	1103 ± 216
Net Energy ^5,7^		761					

^1^ Cultivar Finola, ^2^ Cultivar Armanca, ^3^ Cultivar Unika-b, ^4^ Average of 6 cultivars, ^5^ Energy is expressed as kJ per 100 g of DM, ^6^ Metabolisable Energy, estimated according to Axelsson, 1941 [35], ^7^ Net Energy for lactation (sheep), estimated according to INRA [16].

**Table 3:** Fatty acid profile (% of total FA) of lipids contained in whole hempseed (HS), hemp cake (HC) and hemp oil (HO).

References	[43]	[43]	[17]	[18]	[19]	[15]	[18]	[44]	[15]	[9]	[45]	[46]	[47]	[15]
Products	HS ^1^	HS ^2^	HS	HS ^3^	HS	HS ^4^	HC ^5^	HC	HC ^4^	HO ^5^	HO	HO	HO	HO
C12:0					0.26	0.11			0.09					
C14:0	0.12	0.07			0.04	0.19		0.07	0.17		0.03		0.03	0.08
C16:0	7.27	7.37	6.47	6.2	5.89	9.10	9.3	4.46	8.77	5.0	6.07	7.9	6.54	7.46
C18:0	3.01	2.67	2.87	2.1	2.05	2.72	3.8	1.76	2.51	2.0	2.38	2.70	2.73	2.50
C20:0	3.93	4.40	0.91			0.91		0.71	0.74		0.87	0.8		1.53
C22:0								0.38			0.34			
C24:0								0.22			0.17			
Others SFA	0.12		0.68					0.06						
Total SFA	14.45	14.51	10.93	8.3	8.24	13.03	13.1	7.66	12.28	7.0	9.86	11.4	9.3	11.57
C16:1	0.24	0.32			0.15	0.17		0.15	0.14		0.14	0.2		0.14
C18:1n-7								0.85						
C:18:1n-9	13.14	13.57	12.06	9.5	10.11	16.14	13.1	8.27	13.83	9.0	10.26	20.3	10.91	13.45
C20:1n-9	0.85	0.93	0.42		0.62	0.49		0.46	0.52		0.40	0.4		
C22:1n-9			0.02		0.28			0.00			0.03			
C24:1n-9								0.10						
Other MUFA	0.46	0.84	1.11		0.15			0.32			0.22			
MUFA	14.45	15.34	13.61	9.5	11.16	16.33	13.1	10.00	14.35	9.0	10.91	20.7	10.91	13.45
C18:2 n6	55.34	55.15	56.2	56.1	56.50	55.59	52.5	59.52	56.98	56.0	55.75	51.3	55.78	54.59
C18:3n-3	15.15	14.74	15.25	22.4	21.15	12.98	19.1	15.85	14.62	22.0	17.37	15.70	20.65	15.83
C18:3n-6			2.97	3.7		1.45	2.2	4.52	1.60	4.0	4.65	0.00		
C18:4n-3			0.89							2.0	1.48			
C20:2n-6								1.38						0.19
C20:3n-3	0.45	0.40						0.05						
C20:5n-3								0.16						
Other PUFA	0.16	0.14			0.05			0.10						
Total PUFA	71.10	70.15	75.5	82.2	77.7	70.02	73.8	81.58	73.2	84	79.25	67	76.43	70.61
n-3 PUFA	15.60	14.89	16.14	22.4	21.15	12.98	19.1	16.06	14.62	24	18.85	15.7	20.65	15.83
n-6 PUFA	55.34	55.15	59.17	59.8	56.5	57.04	54.7	65.52	58.58	60	60.40	51.3	55.78	54.78
n-6/n-3 ratio	3.55	3.70	3.67	2.67	2.67	4.39	2.86	4.08	4.01	2.5	3.20	3.27	2.7	3.36

^1^ Cultivar Fedora 17, ^2^ Cultivar Ferimon, ^3^ Cultivar Armanca, ^4^ Average of 6 cultivars, ^5^ Cultivar Finola.

**Table 4:** Effect of hempseed cake (HC) on milk yield and composition [33].

Groups	HC Dosage (% DM)	Milk Yield (kg/d)	Milk Protein (%)	Milk Fat (%)	Milk Urea (mmol/l)	Protein Efficiency ^1^
Control	0	25.2	3.63	4.31	2.7	0.29
HC14	14.3	28.7	3.61	4.21	3.7	0.26
HC23	22.3	26.8	3.49	4.07	4.4	0.22
HC32	31.8	26.8	3.40	3.89	5.1	0.22
*p*-value ^2^		0.022	0.028	n.s.	<0.001	0.009

^1^ Milk protein yield/crude protein intake, ^2^ n.s. = not significant.

**Table 5:** Effect of hempseed and derivatives on the chemical composition of ewe milk.

References	Treatment ^1^	Dosage (% on DM)	Milk Yield (g/d)	Milk Protein (%)	Milk Fat (%)	Lactose (%)
[18]	CTR	0	728	5.61	7.42	5.20
	HS	6.7	781	5.60	8.12	5.10
	HC	22.6	767	5.62	7.97	4.85
		*p*-value ^2^	<0.05	n.s.	<0.01	<0.05
[19]	I	0	669	5.78	7.45	5.20
	I+ HS	8.3	686	5.61	8.36	5.14
	PTG	0	770	6.11	7.39	5.02
	PTG + HS	8.3	784	6.15	7.98	5.09
		*p*-value ^2,3^	n.s.	n.s.	<0.01	n.s.
[50]	CTR	0		5.25	6.40	4.69
	HS	5.0		5.17	5.96	5.84
		*p*-value ^2^		n.s.	n.s.	<0.01

^1^ CTR = control; HS = hempseed; HC = hemp cake; I = indoor feeding system; PTG= part-time grazing feeding system; ^2^ n.s. = not significant, ^3^ *p*-value: effect of HS supplementation.

**Table 6:** Effect of hempseed and derivatives on the fatty acid profile (% of total FA) of ewe milk.

References	Treatment ^1^	Dosage (% on DM)	PUFA ^2^	n-3	n-6	n6/n3	CLA
[18]	CTR	0	6.98	1.99	3.81	1.91	1.18
	HS	6.7	9.85	3.34	4.12	1.23	2.39
	HC	22.6	10.60	2.94	4.35	1.48	2.81
		*p*-value ^3^	<0.001	<0.01	n.s.	<0.01	<0.01
[19]	I	0	5.63	1.31	0.30	5.63	1.13
	I+ HS	8.3	7.92	1.67	0.35	7.92	2.29
	PTG	0	7.40	2.06	0.39	7.40	2.12
	PTG + HS	8.3	9.11	2.09	0.56	9.11	2.90
		*p*-value ^3,4^	<0.001	<0.01	<0.01	<0.01	<0.01

^1^ CTR = control; HS = hempseed; HC = hemp cake; I = indoor feeding system; PTG= part-time grazing feeding system; ^2^ PUFA = polyunsaturated fatty acid. ^3^ n.s. = not significant, ^4^ *p*-value: effect of HS supplementation.

**Table 7:** Effect of hempseed oil on milk yield and quality of goats.

References	Treatment	Dosage (% on DM)	Milk Yield (g/day)	Milk Fat (%)	Milk Protein (%)
[47]	CTR	0	1280	2.70	3.16
	Hemp Oil	4.7	1330	3.59	3.28
		*p*-value ^1^	n.s.	<0.001	<0.05
[63]	CTR	0		3.39	
	Linseeds	9.3		3.73	
	Hempseed	9.3		3.69	
		*p*-value		0.013	

^1^ n.s. = not significant.

**Table 8:** Effect of hempseed oil (HO) on fatty acids, cholesterol and vitamin A of goat milk.

Reference	Treatment	Dosage (% on DM)	PUFA	n-3	n-6	CLA ^1^	Cholesterol (mg/100 g)	Vitamin A (μg/mL)
[47]	CTR	0	5.30	1.35	2.57	0.49	14.63	0.167
	HO	4.7	7.69	1.57	2.94	2.14	11.83	0.151
		*p*-value ^2^	<0.001	n.s.	0.10	<0.001	n.s.	n.s.

^1^ cis-9, trans-11 CLA, ^2^ n.s. = not significant.

## Data Availability

Not applicable.
